# Light Wanted

**Published:** 1888-01

**Authors:** 


### LIGHT WANTED.

A. J., æt. forty-four, painter by trade, nervous and bilious temperament; black hair and eyes; five feet, eleven; weight one hundred and fifty-eight. On first going to sleep has a sensation: "something strikes the chest, arresting the breath, goes to the throat, and there is a choking and severe struggle to get his breath, which arouses him." This is repeated from two to ten times before he gets his sleep, which is natural enough the rest of the night. If aroused during his sleep for any cause has no trouble on again going to sleep. In lieu of this choking has several times had a sensation as of a "hot flash," a streak of lightning going from chest to the great toe of one limb. Has the above symptoms during months not engaged in painting. Has been afflicted for past two years. A short time before he had his trouble, while engaged in painting a mast, the rope fastenings gave way and let him down (in his chair) rapidly; was not hurt, but frightened when he looked up and realized what had happened. He does not know that this had anything to do with his affliction. Is nervous; easily excited, of a quiet, retiring disposition, and complains of nothing else; antecedents good. Lach. relieved a little for over three nights. Ignat. relieved somewhat his nervousness. A medical friend suggested Agancus, which did no good. Now, if some of the readers of your most excellent journal will help me to cure this case, shall be grateful.

ENQUIRER.

